# Emerging Gene Therapy Approaches in the Management of Spinal Muscular Atrophy (SMA): An Overview of Clinical Trials and Patent Landscape

**DOI:** 10.3390/ijms241813743

**Published:** 2023-09-06

**Authors:** Aleksei S. Ponomarev, Daria S. Chulpanova, Lina M. Yanygina, Valeriya V. Solovyeva, Albert A. Rizvanov

**Affiliations:** Institute of Fundamental Medicine and Biology, Kazan Federal University, 420008 Kazan, Russia; alekssponomarev@kpfu.ru (A.S.P.); daschulpanova@kpfu.ru (D.S.C.); lmyanygina@stud.kpfu.ru (L.M.Y.); vavsoloveva@kpfu.ru (V.V.S.)

**Keywords:** spinal muscular atrophy, SMN, gene therapy, clinical trials, patent landscape

## Abstract

Spinal muscular atrophy (SMA) is a rare autosomal recessive neuromuscular disease that is characterized by progressive muscle atrophy (degeneration), including skeletal muscles in charge of the ability to move. SMA is caused by defects in the SMN1 gene (Survival of Motor Neuron 1) which encodes a protein crucial for the survival and functionality of neuron cells called motor neurons. Decreased level of functioning SMN protein leads to progressive degeneration of alpha-motor neurons performing muscular motility. Over the past decade, many strategies directed for SMN-level-restoration emerged, such as gene replacement therapy (GRT), CRISPR/Cas9-based gene editing, usage of antisense oligonucleotides and small-molecule modulators, and all have been showing their perspectives in SMA therapy. In this review, modern SMA therapy strategies are described, making it a valuable resource for researchers, clinicians and everyone interested in the progress of therapy of this serious disorder.

## 1. Introduction

Spinal muscular atrophy (SMA) is a disease caused by the deficiency of SMN (Survival of Motor Neuron) protein that leads to spinal motor neuron degeneration and progressive muscle atrophy drawing to scoliosis, paralysis and death from respiratory failure. In the most severe cases, onset is prenatal and lethal; in other cases, symptoms start from 6 months [1]. SMA is inherited in an autosomal-recessive way, meaning that both parents are either carriers of defective gene *SMN1*, or it is absent. Mutant protein is unable to be involved in pathway sets that are crucial for cell survival and development interactions. The frequency of heterozygotic carriers in the population may vary in a range of 1:40–80 among different ethnic groups [2], which according to statistics leads to 1 in 10,000 of newborns with SMA [3]. Five types of SMA have been identified, depending on the severity of the course and time of clinical manifestation.

SMA0 is the most severe fatal congenital form with onset at prenatal stages. The fetus moves less in the womb; so, the child is often born with joint deformations (contractures). Newborns are unable to swallow and breathe independently and demise within weeks. Abnormalities in cardiac and gastrointestinal development and autonomic dysregulation have been reported [4].

SMA1 (OMIM 253300), also known as Werdnig-Hoffman disease [5], makes about 55% of all SMA cases [6]. It is also a severe infantile form with a manifestation several months after birth (before 6 months) and is characterized by hypotonia, poor head control and reduced or absent tendon reflexes, heart defects and respiratory failure. Infants have breathing difficulties and their life span is shortened to 18 months in 95% of cases without treatment due to respiratory complications [7].

SMA2 (OMIM 253550) is a mild-to-moderate form with onset during infancy, resulting in children that never walk independently; symptom manifestation starts between 6 and 18 months of age. Respiratory dysfunction is common, as the result of developing scoliosis and weakened intercostal muscles. Type II SMA patients develop hand tremors, contractures, and occasionally ankylosis of the mandible. The expectancy of life is reduced compared to healthy individuals but variable from 2 to 40 years among patients [8].

SMA3 (OMIM 253400), Kugelberg–Welander disease [9], has a clinical manifestation beginning by 1.5–3 years of age. Muscle weakness and loss of mobility develop with age. Patients retain the ability to walk, but with certain difficulties due to muscle atrophy. Muscle hypotonia of varying degrees and weakness are manifested. Life expectancy is not affected by the disease, but it severely impairs its quality [10].

**Figure 1:**
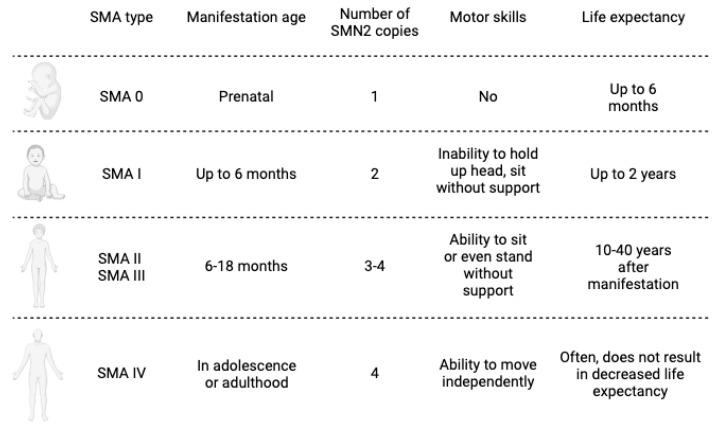
Schematic representation of the pathological types of SMA.

SMA4 (OMIM 271150) is the mildest form, occurring in adults after 18 years of age and presenting with mild proximal limb weakness. The extremely rare form of the disease occurs after 30 years of age. Patients retain motor function and have a normal life expectancy [11] (Figure 1).

The *SMN* genes are localized to the 5q13 region that contains inverted repeats with several copies of the gene [1]. The telomeric copy *SMN1* gene has nine exons producing functional 294-aminoacids 38 kDa SMN protein, usually localized in both the cytoplasm and nucleus, in the Gemini of Coiled bodies compartment. They form Cajal bodies containing high concentrations of small ribonucleoproteins (snRNPs) with pre-mRNAs [12]. SMN contains highly conserved domains essential for its functions in the cell. Mutations in these domains of *SMN1* lead to the synthesis of an inefficient protein.

The centromeric *SMN2* gene is a paralog of the *SMN1* gene and is almost identical to *SMN1* but has five nucleotide changes, one of them causes skipping of exon 7 in 90% of the transcripts in alternative splicing [13]. *SMN2* is 875 kb away from *SMN1* and is formed by a duplication of the ancestral gene unique to the human lineage [14]. Lack of fully functional SMN protein, at least from one copy of the *SMN1* gene, causes SMA manifestation; nevertheless, the number of ~10% full-exonic transcripts from *SMN2*, which are often present in the genome in more than one copy, are enough to prolong and postpone motor neuron degeneration to some extent. The more *SMN2* copies a patient has, the more it compensates the absence of *SMN1*. Thus, in rare cases, individuals with six or more copies have the lightest manifestation after 30 years of age, appearing in mild muscle weakness and preserving full mobility. Most patients of type I SMA carry one or two *SMN2* copies [15]. The number of copies of *SMN2* genes has a strong correlation with disease manifestation; however, some studies show that the *SMN2* copy number is not always a definite index of severity, especially in SMA patients retaining one *SMN1* allele [16]. While being normally expressed, SMN with point mutations may affect the functionality and stability of the protein, contributing to the disease along with other factors described further [17].

The number of *SMN2* copies and the total number of full-length transcripts allows one to predict the severity of SMA. However, mutations in several other genes also contribute to the development of the disease. Patients with deletions in the *NAIP* (NLR family apoptosis inhibitory protein) gene showed significantly earlier onset of the disease with more frequent type I and worse prognosis, including a requirement for ventilation support or outcomes with early death [18]. In less severe cases, female patients showed milder disease than male patients, with more delayed onset of symptoms, probably due to higher levels of CTRP3 protein (C1q/TNF-related protein-3), which stimulates SMN protein translation, also due to better mitochondrial biogenesis and protection against oxidative stress [19]. Mutations in the *TLL2* (Tolloid-like protein 2) gene have also been associated with a more severe manifestation of SMA in men [20]. The functionality of a number of proteins, zinc finger protein ZPR1, FMRP (fragile X mental retardation protein), TDP-43 (TAR DNA-binding protein 43), DDX21 (DExD-Box helicase 21) and many others, affects the disease’s manifestation [21,22]. Mitochondrial functionality decreases with age, which also impairs the symptoms of SMA [20].

According to the Biomolecular Interaction Network Database (BIND) and Molecular INTeraction database (MINT), more than 100 proteins potentially interacting with SMN have been found [21]. Thus, a violation of multiple protein interactions may play a role in the pathogenesis of SMA, but in addition to this, other aggravating causes have been found.

A number of microRNAs are known to play an important role in neurodegenerative diseases and the SMN protein is also involved in their production. Animal SMA models showed that the level of miR-183, miR-9, miR-132, miR-206 and a range of other microRNAs is not normal and may contribute to the disease. They are differentially expressed in SMA patients, and their target genes are associated with fundamental regulatory processes such as translation and transcription, protein phosphorylation, cytoskeletal structure, synapse formation and neuronal development. Measuring microRNA levels can serve as a diagnostic marker in evaluating therapeutic interventions [22,23].

The disruption of various networks of interactions leads to the dysregulation of axon development, their growth cones, and leads to motor neuron lesions [24]. The interaction of SMN protein with cytoskeletal beta-actin mRNA is essential for synapse development and preservation. Studies of neuromuscular junctions showed that neurotransmitter release was reduced by ~55% in most muscles affected by SMA, probably as a result of the destruction of the actin cytoskeleton [25]. It was shown that SMN is part of the ribonucleoprotein complex hnRNPR, whose work determines the fate of axons and growth cones of nascent motor neurons [26]. Indeed, in postmortem studies and mice SMA models, there were found anomalous development of neuromuscular junctions, sensor neurons and motor neurons, leading to the conclusion that only spinal nerves are affected in SMA [27]. However, there is evidence that all body organs and tissues suffer to some degree from SMN deficiency, since it is present in most cells and is included in many multimeric complexes required for key reactions. Its deficiency negatively affects not only motor neurons of the central nervous system (CNS), but also other body systems where its transcription occurs in large amounts—in the kidneys, reproductive system, liver, etc. [28]. With the number of studies, the number of reactions involving the SMN protein continues to grow.

One of the major factors in the development of SMA is the activation of p53-mediated apoptosis due to inefficient pre-mRNA processing during splicing. The spliceosome complex of the cell consists of five types of small nuclear uridine-rich RNAs and proteins, forming snRNPs complexes [29]. The presence of SMN is critical as an intermediate between snRNPs for the assembly of spliceosome complexes and their maturation, through which introns are excised [30]. Mutant forms of the protein found in patients with SMA are unable to stimulate splicing [31]. The retention of introns in transcripts has been shown to induce R-loop formation, DNA damage and activate the p53-mediated apoptosis pathway [32]. Accumulation of R-loops is peculiar in SMA patients [33]. Defects in the assembly of the complexes responsible for R-loop resolution are caused by a lack of interaction between the SMN protein and the SETX (senataxin) protein. Unresolved R-loops lead to DNA damage and deceleration of RNA polymerase 2 [34], leading to a progressive increase in R-loops and further damage, while damage of ribosomal DNA alone impairs their synthesis and translation, and provokes the initiation of cell death [34]. Thus, at least the collapse of spliceosome integrity and massive DNA damage result in motor neuron death by p53-mediated cell cycle arrest or apoptosis, preventing correct neuron development. Interestingly, SMN itself can function as an anti-apoptotic factor binding with p53 [35]. At the same time, a decrease in the p53 protein in SMA models somewhat mitigates the loss of neuromuscular junctions [36]. Neighboring cells and astrocytes provide nutrients, oxygen and specific factors to neurons. They have also been implicated in the pathogenesis of SMA. Mice with SMA were introduced with target *SMN1* gene delivery into astrocytes, excluding neurons, and showed reduced cytokine levels, longer lifespan and improved motility [37].

## 2. Traditional Therapies for SMA Treatment

Traditional therapies for SMA use a variety of approaches to alleviate symptoms and improve patients’ quality of life. These methods, which were widely used before the advent of modern gene therapy strategies, include physical therapy, ergotherapy, and other forms of rehabilitation [38]. Traditional methods are most commonly used in SMA3 and SMA4.

Physical therapy includes exercises and techniques to strengthen muscles, improve mobility and flexibility, and maintain optimal posture and body position. Individual programs are developed for each patient, taking into account the degree of progression of SMA and the peculiarities of their physical condition. Ergotherapy is aimed at helping patients with SMA to perform daily tasks and maintain their independence. Ergotherapists use a variety of methods and techniques to help patients improve their self-care skills, move more efficiently and adapt to the changes caused by SMA. In addition, supportive therapy for SMA focuses on alleviating symptoms and preventing complications. This may include medications to manage pain symptoms, spasms, and other related problems, as well as dietary recommendations [39].

Since 2007, there are treatment standards for SMA, which include addressing respiratory failure (respiratory support) and the prevention of lung infections, nutritional support, orthopedic aspects (especially regarding scoliosis reconstruction), and palliative care issues. New treatments for SMA, currently in various stages of preclinical and clinical research, are also being explored. Methods under development vary in route of administration, dosing frequency, and mechanisms of action, which include neuroprotection, improvement of muscle strength and function, and various methods of modulation of full-length SMN protein levels [40]. For example, valproic acid (a histone deacetylase inhibitor) was shown to increase SMN protein expression multiple times in fibroblasts obtained from patients with SMA and subsequently increase the average survival rate of SMA model mice (mice had increased SMN protein in the spinal cord and improved damage of muscles and neurons) [41]. However, side effects, mainly weight gain, were found in clinical trials, which were associated with changes in motor function and refusal of further testing of this drug [42].

Myostatin inhibition can potentially increase muscle mass in diseases inducing muscle atrophy, but such a treatment has not shown strong positive results in human trials. Although, in combination with other protein pathways corrections, it may be used for a few specific types of neuromusclular diseases [43]. Drugs that sensitize calcium sensor regulatory complex in sarcomere via slowing down the release of Ca^2+^ have a possibility of ameliorating SMA symptoms [44]. Neuroprotectors riluzole [45], involved in glutamate signalling and intracellular Ca^2+^ levels regulation, and olezoxime [46], a lipophilic molecule showing regenerating effects, were ineffective enough to be excluded from clinical development.

Albuterol is a β2-adrenergic receptor agonist used primarily in asthma, but it has been shown to increase full-length SMN transcript levels in cell lines derived from patients with severe SMA. A small study in patients with SMA2 and SMA3 showed a statistically significant increase in muscle function; however, there are no data from larger placebo-controlled studies suggesting widespread use of albuterol in clinical practice for SMA [41].

Thus, the need for gene-therapy approaches is dictated by the unsatisfactory results of classical drugs. Gene therapy consists of delivering the nucleotide sequence of the *SMN1* gene into the patient’s cells and thus restoring its expression. As a result, the cells produce a complete SMN protein, which is necessary for normal motoneuron function.

## 3. Gene Therapy Strategies for SMA

**Figure 2:**
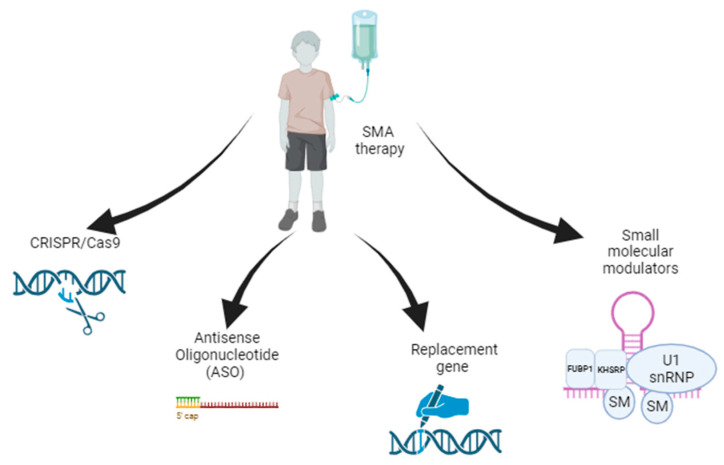
Current therapeutic approaches to SMA.

SMA gene therapy aimed at increasing the level of the functional SMN1 protein can be based on two independent strategies. First is an exogenous *SMN1* gene introduced by a recombinant viral vector encoding a full-length transcript. Second is the increasing of the inclusion of exon 7 in *SMN2* splicing by altering the process [47]. Both of these approaches will be discussed in this section (Figure 2). A summary of the clinical trials described in this and other chapters is given in Table 1.

### 3.1. Gene Replacement Therapy

Adeno-associated viruses (AAVs) are nonenveloped viruses that store genetic information in the form of single-stranded DNA. To date, the use of AAV in clinical drug development has increased considerably because they are capable of transducing dividing and non-dividing cells, and combine low immunogenicity and low pathogenicity with long-term transgene expression in clinical applications. In addition, an important advantage of AAV over other viral vectors for transgene delivery is the minimal risk of insertional mutagenesis, since the virus DNA exists in the host cell as an episome [48]. However, there is also evidence of a minor integration of AAV into human chromosome 19 q13.3-qter, which has been named AAVS1 [49].

In laboratory and clinical practice, more than 12 different AAV serotypes are used, which differ in cell tropism (depending on the type of capsid surface proteins). Since the cells of the nervous system are most severely affected by SMA, AAV9 or rhAAV10, serotypes may be the most effective for use in therapy, which when administered systemically result in high expression in neurons of the motor cortex, cerebellum, substantia nigra and cervical spinal cord [50].

Introduction of AAV9, which encodes the human *SMN* gene, can be effective when injected intramuscularly. A single injection of scAAV9 into the adult mouse calf muscle was shown to mediate widespread motoneuron transduction and lead to an increase in lifespan from 12 days to 163 days in SMA model mice in vivo [51]. However, intrathecal insertion is considered to be the most effective and is used in most studies. For example, injection in the intracerebroventricular (ICV) space of a self-complementary AAV serotype-9 vector expressing the codon-optimized human SMN1 coding sequence (coSMN1) resulted in the restoration of lifespan and growth with a mean lifespan of 346 days in SMA model mice in vivo [52].

Another AAV serotype, rh10 (AAVrh10), discovered more recently, also mediates effective CNS transduction after intravenous injection into mice. A number of studies have shown that intravenous administration of AAVrh10 achieved similar or higher transduction efficiency than AAV9 in all brain regions studied in a mouse model [53]. However, the use of this serotype for SMA therapy has not been investigated at this time.

Intravenous administration of AAV is also often used as an intrathecal administration because AAV9 vectors are able to cross the blood–brain barrier and efficiently transduce motoneurons in the central nervous system [54]. Preclinical studies established raised SMN expression not only in neurons but in tissues outside CNS with a major increase in mice lifespan after 250 days [55].

The promising results of preclinical trials were the basis for the development of Onasemnogen abeparvovec (Zolgensma) based on AAV9, which is now the only approved gene-therapy drug for the treatment of SMA. Onasemnogen abeparvovec, originally known as AVXS-101, delivers a functional *SMN1* gene in a non-replicating adeno-associated virus capsid (scAAV9). The self-complementary feature of the AAV9 vector in combination with the cytomegalovirus hybrid enhancer and the chicken beta-actin promoter ensures rapid and sustained expression of *SMN1* [56].

The vector with *SMN1* is administered intravenously once, and Zolgensma therapy shows the best results, administered soon after birth, before the manifestation of clinical symptoms of SMA, because apparently there is a delay in the development of motor neurons for several months. Patients with already manifested symptoms, show a less pronounced effect after the drug, which, however, is still significant. The main concern, besides the high price of the drug, is safety, since toxic effects on the liver have been shown in some cases [57]. Another issue is the need for use in the first months of life, provided there are no antibodies to the virus serotype, which means available screening programs [58].

During the initial clinical safety trial of AVXS-101-CL-101 (NCT02122952), a group of 15 infants with confirmed SMA1 by genetical analysis was divided into two groups. Three infants received a low dose of abeparvovec onasemnogen (6.7 × 10^13^ vector genes (vg)/kg), while 12 infants received a high dose (2.0 × 10^14^ vg/kg). The first patient experienced increased serum aminotransferase levels, leading to protocol modification for subsequent patients who received prednisolone orally for 4 weeks after drug intake.

The primary outcome of the trial was safety data, specifically any side effects of grade 3 or higher (category 1). The secondary outcome of interest was time to death, or to continuous ventilation, defined as at least 16 h per day for at least 14 days, in the absence of acute disease status or in a condition requiring surgery (category 2). At the 20-month follow-up, all 15 patients were alive without continuous ventilation, which exceeded the expected survival rate based on the natural history of SMA (only 8% of the cohort was expected to be alive). The therapeutic effect of AAV was evaluated based on motor skill development, particularly unassisted sitting, and the CHOP INTEND score [59]. In the high-dose cohort, 11 of 12 patients showed amplified and persistently increased scores on the Children’s Hospital of Philadelphia Infant Test of Neuromuscular Disorders (CHOP INTEND) [60]. Infants who received high-dose AVXS-101-CL-101 were also evaluated at 24 months post-injection. Rapid and significant motor improvements were observed in infants with severe SMA1 who received AVXS-101 at an early age. Specifically, 92% of the patients in the cohort achieved complete control of head retention, were able to sit independently for at least 5 s, and could speak [61].

The efficacy of Onasemnogene abeparvovec was also evaluated for patients with SMN1 deletions and three SMN2 copies develop SMA2. In a Phase III clinical trial (SPR1NT, NCT03505099) of 15 children who received intrathecal injection of Zolgensma within six weeks of administration, all could stand independently up to 24 months, and 14 children could walk independently. No serious adverse events were reported [62]. Another phase 3 study evaluated the effect of Zolgensma administration to children with bi-allelic SMN1 mutations (deletion or point mutations) and one or two copies of SMN2 (STR1VE, NCT03306277). Patients received a single intravenous infusion of abeparvovec onasemnogen (1–1 × 10^14^ vector genomes per kg body weight) for 30–60 min. By age 18 months, 13 (59%) of 22 patients could sit independently for 30 s or longer compared with 23 patients unable to sit independently in the naturally occurring group. An amount of 20 patients (91%) survived without continuous ventilation at 14 months of age (compared with 6 (26%) patients in the naturally occurring group. Side effects found in patients were not considered significant for not recommending treatment [63].

The efficacy of another drug based on self-complementary AAV9 carrying a codon-optimized *SMN* coding sequence (coSMN1) driven by CMV enhancer and chicken β-actin promoter, GC101, will be studied to treat SMA1 (NCT05824169) and SMA2 patients (NCT05901987) in China. Results are to be reported.

### 3.2. Antisense Oligonucleotide Therapy

ASOs are short synthetic nucleotide chains designed to bind selectively through base-mating hybridization to the RNA encoding the protein of interest. Their chemistry entails a modified second-generation 2″-O-(2-meth-oxymethil (2′MOE). ASOs are promising therapeutic agents for a variety of neurodegenerative and neuromuscular disorders, including SMA. To treat SMA, ASOs bind to a specific sequence in intron 7 in a region occupied by a heterogeneous nuclear ribonucleoprotein (hRNP A1/2 proteins) that masks the intron N1 silencer site (ISS-N1). At the ISS-N1 site, ASO promotes the incorporation of exon 7 into the SMN2 pre-mRNA and thus contributes to the increased production of the full-length SMN protein [64]. The therapeutic effect of ASOs was confirmed in SMA transgenic mice injected intraventricularly, which resulted in increased SMN protein expression in the spinal cord, correction of SMA-related molecular and histological pathologies (muscle size, number of motor neurons, and integrity of neuromuscular synapses) and mitigation of phenotype (survival rate and motor activity) [65]. These studies provided a proof-of-concept for the effects of ASOs on alternative splicing of SMN2 and paved the way for subsequent clinical trials.

Nucinersen (IONIS SMNrx, Spinraza) is based on an antisense oligonucleotide developed by Ionis and Biogen and is an FDA-approved drug for the treatment of SMA. Its efficacy has been confirmed for all subtypes of SMA (caused by mutations in chromosome 5q). However, it has been most effective in children with SMA1, increasing their life expectancy and time without artificial lung ventilation [66]. Nucinersen injections are given intrathecally, since ASOs do not penetrate the blood–brain barrier and must be injected directly into the central nervous system. As mentioned above, SMN protein deficiency negatively affects not only motor neurons but also other organ systems. In the case of Nucinersen, they are left without therapeutic effects [40]. The open-label NURTURE phase 2 clinical trial (NCT02386553) investigated the efficacy of Nucinersen for 25 children with genetically diagnosed SMA who first received Nucinersen in infancy in a presymptomatic state. At the time of the last follow-up, the infants were older than the expected age of symptom onset; all 25 participants achieved the ability to sit without support, 23/25 (92%) learned to walk with assistance, and 22/25 (88%) learned to walk independently [67]. Another clinical trial evaluated the efficacy of Nucinersen among 20 participants with infantile-onset SMA symptoms (NCT01839656). Participants received multiple intrathecal loading doses of Nucinersen equivalent to 6 mg or the equivalent dose of 12 mg, followed by maintenance doses of Nucinersen equivalent to 12 mg. An improvement in motor development on the Hammersmith Infant Neurological Examination Section 2 (HINE-2) scale [68] was achieved in 12 (63%) of the 19 participants evaluated [69]. Another phase 1b/2a clinical trial was an open-label, dose-escalating study (3, 6, 9, 12 mg) that enrolled children with late-onset SMA aged 2–15 years. After 3 years of follow-up, The Hammersmith Functional Motor Scale Expanded (HFMSE) improved in 78% of children with type II SMA (mean 10.8 points) and 36% of children with SMA3 (mean 1.8 points), respectively, compared with a 1.7-point decrease in HFMSE in the natural history study of children with SMA2 or SMA3 [70]. The most common side effects of Nucinersen administration included fever, cough, pneumonia, and upper respiratory tract infections (EMBRACE, NCT02462759) [71]. However, the study was not terminated due to no adverse events in any patient.

### 3.3. Small-Molecule Modulators

Another approach for modulating *SMN2* mRNA splicing is to use small-molecule modulators that bind to pre-mRNA and enhance *SMN2* splicing [72].

The only FDA-registered drug in this category is Risdiplam, also known as RO703406 and RG7916. Given orally, it has been demonstrated to have favorable availability to the central nervous system and peripheral tissues, which is a distinct advantage over Nucinersen [73]. Risdiplam was evaluated in patients with SMA1 (FIREFISH study, NCT02913482), in patients with SMA2 and SMA3 (SUNFISH study, NCT02908685), and in patients with presymptomatic symptoms (RAINBOW FISH, NCT03779334).

In part 1 of FIREFISH, a total of 21 patients with deletion of SMN1 in both alleles and two copies of SMN2 were treated for over 1 year. Among them, four patients were administered a low dose of ricodiplam, while the remaining 17 received the dosage currently used in the second part of the study. All patients experienced their initial symptoms between 1 and 3 months after birth, with an average age of 6.7 months at the time of first contact. During the study period, two patients died within the first 12 months, and another patient diseased immediately after the 12-month study. Out of the 17 patients in the highest dosage group, seven were capable of sitting independently for 5 s. Moreover, ten out of these 17 patients achieved a CHOP INTEND score of 40, a milestone that was not observed in untreated patients with SMA1. Furthermore, none of the patients experienced a loss of swallowing ability, nor did they require continuous ventilation beyond the 12-month mark [74].

The SUNFISH Phase 3 study, which included 180 patients with SMA ages 2 and 3, and ages 2 to 25, showed that at 24 months of treatment with riddiplamin, 32% of patients showed improvement (change ≥ 3 on the Motor Function Measure 32 (MFM-32) scale) compared with baseline on the total MFM32 score, with 58% of patients having stabilized disease (change ≥ 0 on MFM-32) [75,76].

In addition, Risdiplam has been shown to be effective in treating non-sitter patients with SMA2 over 16 years of age (NCT04256265). Five patients out of six had clinically meaningful improvements on the Egen Klassifikation 2 scale (>2 points) [77], including the motor (axial and upper limbs), bulbar (speech and swallowing), and respiratory (coughing) domains [78].

Branaplam (known under development code LMI070) is a pyridazine derivative, which also interacts with pre-mRNA of *SMN2* gene and stimulates the inclusion of exon 7 into the final transcript, increasing functional SMN protein quantity in cells [79]. Branaplam is administered orally similar to Risdiplam. The efficacy of Branaplast in children with SMA in a phase 1/2 clinical trial (NCT02268552) has now been completed. However, no data are currently available.

### 3.4. CRISPR/Cas9-Based Gene Editing

There are also genome-editing strategies for correcting the SMA phenotype, for example, using a genome-editing strategy for the Cas9-mediated disruption of splicing-regulatory elements (SREs) located in intron 7 of *SMN2*: ISS (intronic splicing silencer)-N1 and ISS + 100. It was shown that CRISPR/Cas9-based disruption of two SMN2 SREs (ISS-N1 and ISS + 100) resulted in enhanced *SMN2* exon 7 inclusion rates and increased full-length SMN expression in SMA patient-derived iPSCs and motor neuron as well as increased lifespan of the germline-corrected SMA mice to >400 days [80]. Although so far having inconclusive conclusions regarding the possibility and feasibility of introducing this approach in humans, the use of a CRISPR/Cas9-mediated SRE disruption strategy opens the way for further development of effective treatment approaches. In addition, it is also important to determine the efficacy of different delivery options (e.g., AAV, lipid nanoparticles, and exosomes).

### 3.5. Practical Difficulties of Current Treatment Approaches

Despite the positive results of Zolgensma, risks still remain. This drug has been approved for two groups of patients (1, patients with 5q SMA with a bi-allelic mutation in the *SMN1* gene and a clinical diagnosis of SMA1; 2, patients with 5q SMA with a bi-allelic mutation in the *SMN1* gene and up to 3 copies of the *SMN2* gene), but there are no data on age or weight limits. Theoretically, a large number of patients with SMA would be able to receive Zolgensma treatment. However, available clinical trial data cover only patients within the first six months of life with a body weight of less than 8.4 kg, and little is known about the safety and effectiveness of Zolgensma in older or heavier patients [81]. Also, safety and tolerability must be strictly monitored because acute hepatotoxicity and toxicity to sensory neurons have been reported in primates and pigs after intravenous administration of high doses of AAV vectors expressing the human SMN protein [82].

An important problem may be a decrease or lack of efficacy of therapy due to the presence of pre-existing antibodies against AAV9 in the population of patients with SMA [83,84].

Even after all the restrictions are confirmed, a significant problem will remain—the price of this drug—which is about $2 million per single dose of Zolgensma. It is one of the most expensive drugs on the market [81].

Regarding ASOs, promising for the treatment of multiple neurodegenerative diseases, Nucinersen (Spinraza) is currently the first and only approved SMA therapeutic agent for children and adults. Despite its long half-life in the CNS, an obvious disadvantage is the need for four doses followed by three annual maintenance doses, requiring patients to have repeated intrathecal injections [85]. However, if single genotype correction with Zolgensma is not effective, Spinraza treatment remains a treatment option for these patients.

Another problem with drugs that regulate pre-mRNA splicing is the possibility of non-target effects. Risdiplam and Branaplam have been shown to trigger massive perturbations of splicing events, inducing off-target exon inclusion, exon skipping, intron retention, intron removal, and alternative splice site usage [86]. Therefore, more effective dosing regimens need to be identified in order to safely use available therapeutic agents aimed at modulating splicing.

## 4. Methods of Combined Gene Therapy

Comprehensive methods of using gene therapy to treat SMA, such as the combination of Zolgensma and Spinraza, represent a promising approach that can improve efficacy and long-term treatment outcomes. Zolgensma can provide immediate delivery of the *SMN1* gene and increase the overall level of functional SMN protein in motor neurons. Spinraza, in turn, can enhance SMN protein production by activating the *SMN2* gene and improve its distribution across cells. The combined approach may have potential benefits, such as improved treatment efficacy, maximization of motor neuron coverage, and duration of therapy. This could lead to a greater improvement in motor function, increased neuronal survival, and improved quality of life in patients with SMA [87].

However, according to a recent study, the combination of two drugs for SMA therapy may be less effective in treating children with SMA1 compared to Spinraza alone. The authors of the study formed two groups of children with type 1 SMA. The first group received the combined treatment of Spinraza and Zolgensma, while the second group received only Spinraza. There were seven children in the combined group, of whom six started treatment with Spinraza at 2 to 6 months of age and then received Zolgensma. One child in this group was first treated with Zolgensma and then started Spinraza treatment 18 months later. The second group of six children used only Spinraza, which was started at 2 to 6 months of age [88].

However, according to a recent study, the combination of two drugs for SMA therapy may be less effective in treating children with SMA1 compared to Spinraza alone. The authors of the study formed two groups of children with type 1 SMA. The first group received the combined treatment of Spinraza and Zolgensma, while the second group received only Spinraza. There were seven children in the combined group, of whom six started treatment with Spinraza at 2 to 6 months of age and then received Zolgensma. One child in this group was first treated with Zolgensma and then started Spinraza treatment 18 months later. The second group of six children used only Spinraza, which was started at 2 to 6 months of age [89].

Recruitment for the RESPOND [NCT04488133] clinical trial is now underway to evaluate the safety and tolerability and clinical outcomes of Nucinersen therapy in participants with SMA who previously received onasemnogen abeparvovec. The first results will be published in September 2024. Another JEWELFISH study (NCT03032172) is an ongoing study of patients who received Risdiplam after prior SMA treatment, a subset of whom received gene transfer before starting Risdiplam treatment

Thus, the peculiarities of the combination approach are the practical and financial difficulties associated with the use and availability of drugs. Further studies on a larger number of patients, to determine the safety of the combined use of existing drugs for the therapy of SMA are needed.

## 5. Intellectual Property in SMA Therapy: Patent Landscape

Intellectual property (IP) issues are critical aspects of the development and commercialization of SMA therapy. Patent disputes and litigation can arise in a variety of contexts, such as competing claims for fundamental technologies, the use of transgene delivery vectors, or their functional elements. Understanding the state of the art is necessary to provide a clear path to market-promising therapies and to maintain incentives for innovation. The most important patents in the field of SMA therapy development are presented in Table 2.

For example, the invention WO2010129021A1 describes standard recombinant AAV (rAAV) vectors and recombinant self-complementary (scAAV) vectors capable of delivering genes into the CNS for the treatment of neurodegenerative diseases such as SMA. Studies were conducted in a mouse model of SMA, where it was shown that after administration of AAVhSMN1, animal survival increased from 15 to 50 days, an increase of 233%. The expression level of *hSMN1* gene in the spinal cord and the subcellular distribution of hSMN protein in spinal cord motoneurons of treated and untreated SMA mice were evaluated. The cross-sectional area of myofibrils of muscle groups in treated and untreated mice was estimated, and the size distribution of muscle fibers was similar between the SMA model and wild-type mice. Expression of scAAV8-hSMN increased the number of motor neurons and improved neuromuscular junctions (NMJ) in SMA mice.

The patent [WO2017100671A1] describes in detail amino acid sequences, such as QAVRTSL (SEQ ID NO: 37), which may be a useful part of the capsid protein of the AAV vector. There are also sequences of 11 contiguous amino acids with specific properties and configurations. The invention encompasses a pharmaceutical composition comprising one or more disclosed AAV vectors and one or more pharmaceutically acceptable carriers. A method of delivering a nucleic acid to a targeting medium of a subject using an AAV is described. The vector can target a variety of tissues including the heart, nervous system, or a combination thereof. The patent discusses the use of recombinant AAVs (rAAVs) for in vivo gene transfer.

The invention [WO2019147960] shows the prognostic efficacy of the biomarker pNF-H and pNF-L in plasma, and their correlation with methods for the diagnosis and prognosis of SMA such as HINE-2 and CHOP INTEND. pNF-H was evaluated under various conditions in the CHERISH, ENDEAR, NURTURE, and EMBRACE clinical trials.

The invention [WO2018187209] describes the potential use of ALK4:ActRIIB for the treatment of SMA. For example, it has been shown that the soluble ALK4:ActRIIB heterodimer can be used to prevent or reduce the severity of muscle and bone loss in a model of SMA. Moreover, treatment with ALK4:ActRIIB increased muscle strength in patients with SMA. As described herein, ALK4:ActRIIB heterodimeric proteins are unique antagonists of TGF-beta superfamily ligands, exhibiting a different ligand binding profile/selectivity compared to the corresponding ActRIIB and ALK4 homodimers. In particular, the typical ALK4:ActRIIB heterodimer exhibits increased binding to activin B compared to either homodimer, retaining strong binding to activin A, GDF8 and GDF11, as observed with the ActRIIB homodimer, intermediate binding to BMP6, and exhibiting significantly reduced binding to BMP9, BMP10 and GDF3.

Thus, these results demonstrate that ALK4: ActRIIB heterodimers are more selective antagonists (inhibitors) of certain ligands of the TGF-beta superfamily compared to ActRIIB homodimers. It has been found that antagonists of ALK4: ActRIIB signaling (e.g., signaling mediated by one or more of the activins, GDF11, GDF8, BMP6, GDF3, and BMP10) can be used for the treatment of SMA.

The prior Zolgensma invention was developed by AveXis, which was subsequently acquired by Novartis and converted into its Novartis Gene Therapies division [WO2019094253]. The constructs required to assemble the viral construct are described, including the *SMN* gene plasmid, helper and capsid plasmids. Also described is the process of making viral particles in HEK293 cell culture in a bioreactor. The patent discloses key indications for a finished drug substance comprising AAV9-SMN1.

The patent [WO2020113034] describes methods and compositions for treating type II or III SMA using intrathecally administered AAV9 carrying the *SMN1* transgene. The AAV9 vector construct includes specific elements for efficient gene expression. Patient criteria, steroid dosing and treatment ranges, and monitoring strategies using scales such as Hammersmith Functional Motor Scale-Expanded (HFMSE) and Bayley Scales are defined. The patent also discusses possible combination therapies and details the pharmaceutical formulation of the AAV9 viral vector.

The invention WO2008095357 provides the use of a recombinant genetic vector comprising at least one copy of an expressed gene encoding StatδA, and comprising at least one copy of a Statδ inhibitor, for the development of a drug for the treatment of SMA or other SMN deficiency in humans. The Statδ activator can be the following: interferon-alpha (IFNα); interleukins IL-2, IL-3, IL-5, IL-6, IL-7, and IL-15; granulocyte/macrophage colony-stimulating factor (GM-CSF); growth hormone (GH); epidermal growth factor (EGF); erythropoietin (EPO); prolactin (PRL); thrombopoietin (TRP); trichostatin A (TSA); aclarubicin; sodium vanadate; and combinations thereof. A viral vector, which is an adenovirus, adeno-associated virus, herpesvirus, or lentivirus vector, is described in the invention.

The invention [WO2018160585] describes the vector AAVhu68.CB7.CI.hSMNlco.RBG and methods of using it to treat spinal muscular atrophy. The AAVhu68 capsid was chosen because of its specific properties, which include a high level of tropism into motoneurons in the CNS. Utilization of this vector by intrathecal administration to mice allowed successful restoration of SMN expression in various CNS tissues. The treated animals showed improved motor function, weight gain and survival compared to the untreated control group.

The patent [WO2014184562] describes an invention comprising the use of lentiviral vectors designed to treat diseases associated with neuronal degeneration, such as amyotrophic lateral sclerosis (ALS) and SMA. The vectors are designed for targeted delivery to motor neurons by incorporating antibodies that bind to presynaptic terminal receptors at neuromuscular junctions and a fusogenic protein that promotes endosomal release. This approach increases vector specificity by allowing transduction preferentially in motor neurons, while providing a minimally invasive way to deliver therapeutic agents to hard-to-reach areas such as the CNS. In vivo studies have demonstrated successful transduction and tropism to motor neurons when the vector is administered intramuscularly, indicating the potential of this technique for gene therapy applications.

The patent [WO2019118734] also relates to compositions and methods for treating motor neuron diseases, in particular spinal muscular atrophy (SMA) and amyotrophic lateral sclerosis (ALS). The invention is based on recognizing the genetic and functional links between these diseases, thereby providing a general approach to treating them. The disclosure describes a method for treating motor neuron diseases by administering nucleic acid molecules encoding modulators of the heat shock protein Hsp70, such as mutant Hspa8, with specific characteristics such as missense mutations or altered domains. Heat shock proteins are important multifunctional proteins that play a role in polypeptide chain folding, cellular defense, etc. These mutant proteins have potential advantages such as higher microautophagy activity compared to wild-type counterparts. In addition, the patent details the use of modulators, such as inhibitors or nucleic acid molecules, to target Hsp70 family proteins. The target for such treatment is the central nervous system, including the spinal cord, which may include various methods of administration such as intrathecal injection, oral administration, intravenous infusion, and the like. The invention provides for the use of recombinant AAV vectors to deliver nucleic acid molecules to the nervous system. The patent emphasizes the potential relevance of this approach for the treatment of various motor neuron diseases and provides experimental results demonstrating the therapeutic effect of mutant Hspa8 in a model of SMA. Notably, the patent thoroughly describes the scientific data to suggest a new way to treat these severe neurodegenerative diseases.

The patent [WO2012160130A1] provides methods for treating neuromuscular diseases by administering ERK inhibitors. These inhibitors, particularly MEK1/2 inhibitors, interfere with the MEK ERK1/2 signaling pathway necessary for cellular communication. This approach involves reducing the expression of *MEK1*, *MEK2*, *ERK1* and *ERK2* genes using small molecules or nucleic acid molecules such as siRNA, shRNA or antisense oligonucleotides. The patent emphasizes the potential for the oral administration of ERK inhibitors to treat these diseases, including potential combinations with other active agents such as siRNA, shRNA or antisense compounds targeting the deficient *SMN1* gene product, offering a promising strategy to combat the loss of motor function caused by SMN deficiency. As in the previous invention, the use of substances associated with complex molecular cellular functioning that involves many proteins is hypothesized to affect the body in a negative manner. Therefore, more research is needed for the inventions WO2019118734 and WO2012160130A1.

The invention [US20190136192] comprises modified mesenchymal stem cells (MSCs) for enhancing tissue regeneration and combinations of mesenchymal stem cells together with extracts and/or products derived from said mesenchymal stem cells, which are used to prevent, inhibit the progression and/or reversal of spinal muscular atrophy. A special feature of the present invention is the use of MSCs, since at present, the effect and safety of these cells have not been fully investigated and there are risks of cancer.

The patent [WO2019011817] provides a recombinant adeno-associated viral vector (rAAV) comprising a single-stranded genome and an AAV9 or AAVrh10 capsid for delivery of the human *SMN* gene. The expression cassette of the vector contains a promoter that functions in lower motor neurons or glial cells of the spinal cord to provide cell-specific activation of the *SMN* gene. The single-stranded genome is outperformed by the traditional self-complementary genome in increasing survival in animal models of SMA. The patent highlights the optimized design, sequences and configurations. In experiments, administration of 8 × 10^12^ copies (vg/kg) of the AAV9-hSMN1 single-chain vector resulted in a significant increase in the lifespan and growth of SMA mice, with 40% of animals achieving a survival rate of 245 days, significantly better than previous studies with self-complementary AAV vectors. This demonstrates the efficacy of single-chain AAV vectors in the treatment of SMA.

The invention [WO2021246909] provides a wild-type and codon-optimized sequence using the GeneBeam algorithm encoding the SMN1 protein. An expression cassette and a recombinant AAV9 vector based on it are also described. The efficiency of the developed construct was evaluated at the mRNA and protein levels after the transfection of the HEK293 and HSMC cell lines.

The patent [WO2021030766] describes methods and compositions for treating SMA using combination therapy. These methods include administering a small molecule that improves SMN function along with a recombinant nucleic acid encoding SMN1 or an antisense oligonucleotide (ASO) that increases full-length SMN2. The patent proposes various combinations and sequences of drug administration to treat different degrees of disease severity and increase intracellular SMN activity in motor neurons. The therapy utilizes splicing modulators, HDAC inhibitors, and molecules that modulate mRNA-decapping enzymes as small molecules to enhance SMN function. In addition, the use of recombinant nucleic acids, particularly in viral vectors such as recombinant AAV and ASOs targeting SMN2 mRNA splicing, appears as a promising approach to treat SMN. The patent emphasizes the flexibility of these combination therapies, both when administered simultaneously and sequentially, to provide effective treatment options for patients with varying degrees of severity of SMA.

Thus, the importance of solving the challenges and intellectual property issues for the successful development and implementation of gene therapy is paramount in the treatment of SMA. Understanding the technical level is integral to achieving a balanced approach in fostering innovation and commercialization in this area, thereby providing better pathways for the development of SMA therapies.

## 6. Conclusions and Future Prospects

The way to develop effective therapies for spinal muscular atrophy has witnessed remarkable progress, driven by advances in genetics, molecular biology, and biotechnology. This multi-faceted approach involves understanding the genetic basis of SMA, developing gene therapies, exploring innovative drug delivery methods, optimizing treatment protocols, and addressing intellectual property challenges. The amalgamation of these efforts has ushered in a new era of hope for patients and their families.

SMA, once a dire diagnosis, has seen transformative breakthroughs with the advent of gene therapies. Prominent among these is Zolgensma, a groundbreaking gene therapy that addresses the underlying genetic defect by delivering a functional *SMN1* gene. The clinical success of Zolgensma underscores the potential of gene therapies in treating genetic disorders at their root cause. Complementing this approach is the utilization of alternative therapies like Spinraza, which modulates the activity of SMN2 to increase SMN protein production. The combination of such therapies exhibits promise in further enhancing treatment efficacy, although ongoing research is essential to fully comprehend their synergistic effects.

The evolution of treatment strategies is mirrored by the challenges faced in gene delivery methods. Innovations such as AAV-based vectors have paved the way for efficient and targeted gene delivery to motor neurons. The utilization of AAV serotypes with enhanced tropism for the central nervous system holds the potential to amplify therapeutic impact. Moreover, novel avenues such as RNA-based therapies and small molecules provide diversified approaches to address the complexities of SMA, offering a more comprehensive treatment landscape.

The unfolding landscape of SMA treatment requires an interdisciplinary approach, necessitating collaboration between researchers, clinicians, pharmaceutical companies, and regulatory bodies. Clinical trials play a pivotal role in validating the safety and efficacy of these therapies, paving the way for their approval and widespread availability. Rigorous monitoring, data analysis, and real-world evidence generation are integral to ensuring the long-term benefits and safety of these treatments.

Intellectual property, too, plays a pivotal role in this journey. Patents safeguard the innovative technologies and methodologies that underpin these therapies, offering protection and incentivizing further research and development. The patent landscape is intricate, ranging from vector optimization and gene sequences to combination therapies and delivery methods. Balancing the need for intellectual property protection with fostering innovation and equitable access is a complex challenge that requires careful consideration.

As the field continues to advance, challenges remain, such as refining treatment protocols for different SMA subtypes, optimizing combination therapies, addressing potential long-term effects, and ensuring global accessibility. Collaboration among researchers, healthcare professionals, policymakers, and patient advocacy groups is essential to navigate these challenges and usher in an era of more accessible, effective, and personalized SMA therapies.

In conclusion, the remarkable strides made in understanding the genetic underpinnings of SMA and the innovative approaches to therapy development have transformed SMA from a previously devastating diagnosis to a condition with newfound hope. While challenges persist, the synergy of scientific discovery, technological innovation, and collaborative effort fuels the optimism that SMA can be effectively managed and treated, ultimately improving the lives of those affected by this condition.

## Figures and Tables

**Table 1:** Clinical trials of gene preparations for SMA therapy.

Name of Drug, Company	NCT Number	Phase	Participants Number	Summary Description	Status
Onasemnogene Abeparvovec (Zolgensma) by Novartis Gene Therapies	NCT02122952	1	15, with age <6 months	Dose-escalation trial for safety and efficacy evaluation of intravenous delivery of AVXS-101-CL-101 as a treatment of spinal muscular atrophy Type 1 (SMN1).	Completed 15 December 2017
	NCT03381729	1	32, with age ≥ 6 and <24 months; ≥24 and <60 months	Evaluation of the safety and tolerability of intrathecal administration in infants and children with SMA with bi-allelic deletion in SMN1, with 3 copies of SMN2 and deletion of SMN1. Single-dose administration.	Completed 18 November 2021
	NCT03306277	3	22, with age <6 months	Open-label, single-arm, single-dose trial, intravenous administration of onasemnogene abeparvovec-xioi in SMA Type 1 participants.	Completed 12 November 2019
	NCT03461289	3	33, with age <6 months	Open-label, single-arm, single-dose, trial in patients with SMA Type 1, with bi-allelic pathogenic mutation of SMN1 and 1 or 2 copies of SMN2.	Completed 11 September 2020
	NCT03837184	3	2, with age <6 months	Open-label, single-arm, single-dose, efficacy trial in patients with SMA Type 1, with bi-allelic pathogenic mutation of SMN1 and 1 or 2 copies of SMN2.	Completed 29 June 2021
	NCT03505099	3	30, with age <6 weeks	Open-label, single-arm, single-dose safety and efficacy trial in patients with SMA with bi-allelic deletion of SMN1 and 2 or 3 copies of SMN2.	Completed 15 June 2021
	NCT03421977		13, Child, Adult, Older Adult	Long-term, safety follow-up study of patients in the NCT02122952 gene replacement therapy.	Completion estimated December 2033
	NCT04042025		85, Child, Adult, Older Adult	Long-term, safety and efficacy follow-up study of patients in the AVXS-101 gene replacement therapy.	Completion estimated 29 December 2035
Nusinersen (spinraza), by Biogen	NCT01494701	1	28	Evaluation of the safety, tolerability, and pharmacokinetics of a single dose of nusinersen (ISIS 396443) administered intrathecally to participants with SMA.	Completed 31 January 2013
	NCT01780246	1	18	Safety and tolerability examination of ISIS 396443 administered intrathecally to participants SMA who previously participated in NCT02865109. Examination of the plasma pharmacokinetics of a single dose administered intrathecally to participants with SMA who previously participated in NCT02865109.	Completed 28 February 2014
	NCT01839656	2	21	Clinical examination of efficacy, safety and tolerability of multiple doses of nusinersen administered intrathecally to participants with Infantile-Onset SMA and cerebral spinal fluid and plasma pharmacokinetic examination.	Completed 21 August 2017
	NCT01703988	1, 2	34	Testing safety, tolerability, and pharmacokinetics of escalating doses of nusinersen administered into the spinal fluid either 2 or 3 times in participants with SMA.	Completed 31 January 2015
	NCT02052791	1	47	Testing safety, tolerability, and cerebrospinal fluid and plasma pharmacokinetics in participants with SMA who previously participated in NCT01703988 or NCT01780246.	Completed 31 January 2017
	NCT02193074	3	122	Examination of clinical efficacy, safety and tolerability of nusinersen administered intrathecally to participants with infantile-onset SMA.	Completed 21 November 2016
	NCT02292537	3	126	Examination of clinical efficacy, safety and tolerability of nusinersen administered intrathecally to participants with later-onset SMA.	Completed 20 February 2017
	NCT02386553	2	25	Examination of the efficacy and effects of multiple doses in preventing or delaying the need for respiratory intervention or death in infants with genetically diagnosed and presymptomatic SMA.	Completion estimated 27 January 2025
	NCT04089566	3	145	Examination of clinical efficacy, safety and tolerability of nusinersen in higher doses to participants with SMA.	Completion estimated 2 August 2024
	NCT04729907	3	172	Evaluation of the long-term safety, long-term efficacy and tolerability of nusinersen administered intrathecally at higher doses to participants with SMA who previously participated in NCT04089566.	Completion estimated 30 May 2026
	NCT02594124	3	292	Evaluation of the long-term safety, long-term efficacy and tolerability in participants who previously participated in investigational studies of nusinersen.	Completion estimated 29 August 2023
	NCT04488133	4	60	Evaluation of the clinical outcomes following treatment in participants with SMA.	Completion estimated 4 September 2024
Risdiplam (Evrysdi), by Hoffmann-La Roche	NCT02240355	1	9	Multicenter, randomized, double-blind, 12-week, placebo-controlled multiple dose study will investigate the safety and tolerability of RO6885247 in adult and pediatric patients with SMA. The study was put on hold and eventually terminated.	Completed July 2015
	NCT02913482	2, 3	62	Open-label, multi-center clinical study to assess the safety, tolerability, pharmacokinetic, pharmacodynamics, and efficacy of Risdiplam in infants with Type 1 SMA. The study is divided in two parts.	Completed 14 November 2019 Completion estimated 17 November 2023
	NCT02908685	2, 3	231	Multi-center, randomized, double-blind, placebo-controlled study to assess the safety, tolerability, pharmacokinetics, pharmacodynamics, and efficacy of risdiplam in adult and pediatric participants with Type 2 and Type 3 SMA. The study consists of two parts.	Completed 6 September 2019 Completion estimated 2 September 2023
	NCT03032172	2	174	Multi-center, exploratory, non-comparative, and open-label study to investigate the safety, tolerability, PK, and PK/PD relationship of risdiplam in adults, children and infants with SMA previously enrolled in NCT02240355 or previously treated with nusinersen, olesoxime or AVXS-101.	Completion estimated 27 December 2024
	NCT03779334	2	25, age up to 6 weeks	A global study of oral risdiplam in pre-symptomatic participants with SMA to investigate the efficacy, safety, pharmacokinetics, and pharmacodynamics of risdiplam.	Completion estimated 21 January 2029

**Table 2:** Patent search in the field of SMA therapy.

Title	Applicant	Patent Number	Date of Publication
Gene therapy for neurodegenerative disorders	GENZYME CORPORATION	WO2010129021A1	11 November 2010
ARGETING PEPTIDES FOR DIRECTING ADENO-ASSOCIATED VIRUSES (AAVs)	CALIFORNIA INSTITUTE OF TECHNOLOGY	WO2017100671A1	15 June 2017
Methods of treating spinal muscular atrophy	BIOGEN MA INC. [US].	WO2019147960	1 August 2019
Compositions and methods for treating spinal muscular atrophy	ACCELERON PHARMA INC. [US]	WO2018187209	11 October 2018
Means and method for preparing viral vectors and uses of same	AVEXIS INC.	WO2019094253	16 May 2019
AAV viral vectors and uses thereof	NOVARTIS GENE THERAPIES, INC.	WO2020113034	4 June 2020
Treatment for spinal muscular atrophy	ACADEMIA SINICA	WO2008095357	30 August 2006
Composition useful in treatment of spinal muscular atrophy	THE TRUSTEES OF THE UNIVERSITY OF PENNSYLVANIA	WO2018160585	17 January 2018
Lentiviral vectors with tropism to motor neurons comprising an antibody that binds to a pre-synaptic terminal receptor on the neuromuscular junction and a fusogenic protein	IMPERIAL INNOVATIONS LIMITED	WO2014184562	20 November 2014
Compositions and methods for treating motor neuron diseases	THE TRUSTEES OF COLUMBIA UNIVERSITY IN THE CITY OF NEW YORK	WO2019118734	20 June 2019
ERK inhibitors for use in treating spinal muscular atrophy	UNIVERSITE PARIS DESCARTES; CENTRE NATIONAL DE LA RECHERCHE SCIENTIFIQUE Paris	WO2012160130A1	29 November 2012
Mesenchymal stem cell therapy for spinal muscular atrophy	CELL MEDICINEINC.	US20190136192	9 May 2019
Treatment of spinal muscular atrophy	GENETHON	WO2019011817	17 January 2019
Codon-optimized nucleic acid encoding smn1 protein	Joint Stock Company “Biocad”	WO 2021/246909	9 December 2021
Combination therapy for spinal muscular atrophy	BIOGEN MA INC.	WO2021030766	18 February 2021
